# Identification and Molecular Characterization of *FKF1* and *GI* Homologous Genes in Soybean

**DOI:** 10.1371/journal.pone.0079036

**Published:** 2013-11-13

**Authors:** Fang Li, Xiaomei Zhang, Ruibo Hu, Faqiang Wu, Jinhua Ma, Ying Meng, YongFu Fu

**Affiliations:** 1 MOA Key Lab of Soybean Biology (Beijing), National Key Facility of Crop Gene Resource and Genetic Improvement, Institute of Crop Sciences, Chinese Academy of Agricultural Sciences, Haidian District, Beijing, China; 2 CAS Key Laboratory of Biofuels, Shandong Provincial Key Laboratory of Energy Genetics, Qingdao Institute of BioEnergy and BioProcess Technology, Chinese Academy of Sciences, Qingdao, Shandong, China; Wuhan University, China

## Abstract

In *Arabidopsis*, FKF1 (FLAVIN BINDING, KELCH REPEAT, F-BOX1) and GI (GIGANTEA) play important roles in flowering pathway through regulating daytime *CO* (*CONSTANS*) expression, and such a function is conserved across plants studied. But related reports are limited for soybean. In this study, we cloned *FKF1* and *GI* homologs in soybean, and named as *GmFKF1*, *GmFKF2*, *GmGI1*, *GmGI2*, and *GmGI3*, respectively. *GmGI1* had two alternative splicing forms, *GmGI1α* and *GmGI1β*. *GmFKF1*/*2* transcripts were diurnally regulated, with a peak at zeitgeber time 12 (ZT12) in long days and at ZT10 in short days. The diurnal phases between *GmGIs* transcript levels greatly differed. *GmGI2* expression was regulated by both the circadian clock and photoperiod. But the rhythmic phases of *GmGI1* and *GmGI3* expression levels were mainly conferred by long days. *GmFKFs* shared similar spatio-temporal expression profiles with *GmGIs* in all of the tissue/organs in different developmental stages in both LD and SD. Both GmFKF and GmGI proteins were targeted to the nucleus. Yeast two hybrid assays showed GmFKF1/GmFKF2 interacted with GmGI1/GmGI2/GmCDF1 (CYCLING DOF FACTOR CDF1 homolog in soybean); and the LOV (Light, Oxygen, or Voltage) domain in GmFKF1/GmFKF2 played an important role in these interactions. N-terminus of GmGI2 was sufficient to mediate its interaction with GmCDF1. Interestingly, N-terminus not full of GmGI3 interacted with GmFKF1/GmFKF2/GmCDF1. Ectopic over-expression of the *GmFKF1* or *GmFKF2* in *Arabidopsis* enhanced flowering in SD. Collectively, GmFKF and GmGI in soybean had conserved functional domains at DNA sequence level, but specific characters at function level with their homologs in other plants.

## Introduction

An internal time-keeping mechanism or oscillator known as the circadian clock has been found in most organisms from cyanobacteria, plants to humans [1]. Many physiological and biological behaviors of plants are conferred by the circadian clock, including photosynthesis, leaf movements, hormone production, metabolic activities, growth and development, fitness, and the transition to flowering [2]–[6]. Of which, triggering flowering at the appropriate time is vital for plants to successfully maximize reproduction [7], therefore photoperiodic pathway is an important mechanism of flowering [8], [9].

The photoperiod pathway is controlled by the circadian clock, which is regulated by different components. *FLAVIN-BINDING, KELCH REPEAT, F-BOX1* (*FKF1*) and *GIGANTEA* (*GI*) genes are controlled by the circadian clock. In turn, FKF1 and GI can mediate the stability of some key clock proteins. FKF1 has three functional domains: the LOV domain, the F-box motif, and the Kelch repeats, all of which are highly conserved in two F-box proteins ZEITLUPE (ZTL) and LOV KELCH PROTEIN2 (LKP2) [10]–[12]. FKF1 interacts with GI through the LOV domain to form a complex in a blue-light dependent manner in the late afternoon under LD conditions [13]. Both FKF1 and GI can physically interact with CYCLING DOF FACTOR 1 (CDF1) [13], [14] and result in degradation of CDF1 by ubiquitin–proteasome system [15]. In this process, the F-box motif is involved in formation of the SCF complex, whereas the Kelch repeats are responsible for substrate protein recognition [16], [17]. In addition, the Kelch repeats of FKF1 can interact with CDF1 [14]. The CDF1 protein is a transcription repressor of *CONSTANS* (*CO*) by directly binding to the Dof binding site in the *CO* promoter. Under LD conditions, the timing of *FKF1* and *GI* expression is in phase and sufficient FKF1-GI complex is formed to activate *CO* transcription during the day. Meanwhile, the CO protein is stabilized by light at the end of the day in LD [18]. Such a daytime *CO* expression triggers the expression of the floral integrators *FLOWERING LOCUS T* (*FT*) and *TWIN SISTER OF FT* (*TSF*), which are known as florigens [19]–[21], leading to floral initiation [19], [22]–[25]. In contrast, under SD conditions the expression of *FKF1* and *GI* are out of phase and small amount of FKF1–GI complex is formed in light, causing a low abundance of *CO* mRNA during the day [13]. It has been reported FKF1 and GI regulate flowering time besides through the CO/FT module [10], [26]–[28]. FKF1 may regulate *FT* expression independent of *CO* through the same FKF1-mediated CDF proteins degradation mechanism. FKF1 is also involved in the stabilization of CO proteins in the long-day afternoon and increasing the expression of *FT* [15], [29]. Similarly, GI can activate *FT* expression either through directly binding *FT* promoter or via miR172 [30], [31].

Compared with the photoperiodic flowering regulation, the FKF1 protein has a subtle role in regulation of the circadian clock and may contribute to the ubiquitin-dependent degradation of TIMING OF CAB EXPRESSION 1 (TOC1) and PSEUDO RESPONSE REGULATORS 5 (PRR5) [1], [32]. On the contrary, GI is required for maintaining normal circadian system. Mutations in the *Arabidopsis thaliana GI* gene cause alteration of circadian rhythms in the clock-associated genes, *CIRCADIAN CLOCK ASSOCIATED 1* (*CCA1*) and *LATE ELONGATED HYPOCOTYL* (*LHY*). The circadian period of the clock-controlled gene *cab2::luc* is altered in the *gi* mutant [33]. GI stabilizes ZTL proteins though forming a complex with ZTL and prevents TOC1 and PRR5 from ZTL-dependent degradation in the afternoon [34], [35]. In addition, GI plays multiple roles in plant growth and development [36]–[42]. So far, the information on the isolation and function analysis of *FKF1* in other plant species is limited [43], [44]. However, *GI* is highly conserved in seed plants, such as *Oryza sativa* [45], *Hordeum vulgare* [46], *Triticum aestivum* [47], *Zea mays* [48], *Pisum sativum* [49], *Allium cepa* [43], *Brachypodium distachyon* [50], *Pharbitis nil* [51] and *Lemna gibba* [52].

Soybean (*Glycine max*), a typical short-day plant, is one of the important oil and protein crops in the world. Normal flowering is important for soybean to get yield maximization at a given ecological conditions. Recently, a series of quantitative trait loci and major genes controlling flowering and maturity (E1 to E8 and J) have been identified [53]. Of these, *E1* largely influences the flowering time under field conditions and functions as a flowering repressor [44], [54], [55]. *E2* is identified as a homolog of *Arabidopsis GIGANTEA* [56]. Both *E3* and *E4* loci encode *PHYTOCHROME A* (*PHYA*) homologs, and the functions of *E4* and *E3* are different in response to light quality and photoperiodic length [57]–[59]. In addition, two homologs of *FT* (*GmFT2a* and *GmFT5a*) are found to coordinately promote flowering [60], [61]. *Dt1* gene, corresponding to *Arabidopsis TERMINAL FLOWER 1* gene, has also been identified to condition a change from indeterminate to determinate growth habit [62], [63]. Besides from these genes, the soybean genome contains four Myb transcription factors *LHY1*/*CCA1*-like genes with diurnal rhythm expression [44], [57], [64]. Some photoreceptors like CRYPTOCHROME, ZTL, FKF1 and LKP2 have their homologs in soybean [65]–[67]. Recently, Kim et al. (2012) have identified numerous floral regulatory candidate genes in soybean genome by comparative genomic analysis [68]. However, the functions of many important genes in soybean are waiting to be addressed. In this study, we identified *FKF1* and *GI* homologs in soybean and analyzed their extensive expression patterns, including diurnal rhythms and tissue-organs as well as developmental expression patterns. Transient assay in *Arabidopsis* protoplast and yeast two-hybrid were applied to investigate the protein localization and interaction. Finally, flowering activity of *GmFKF*s and *GmGI*s were evaluated through over-expression analysis.

## Results

### Homologs of *FKF1* and *GI* in Soybean Genome

**Figure 1:**
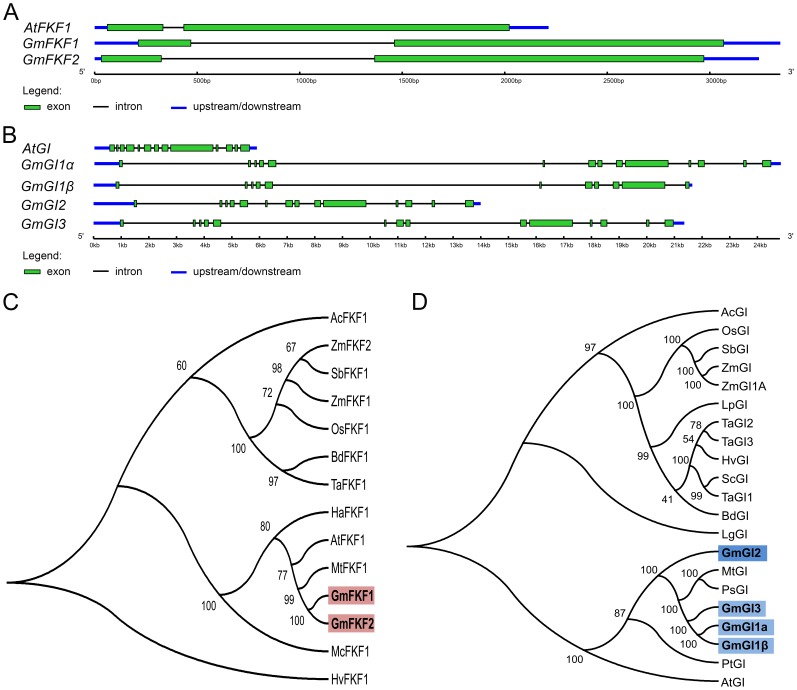
The *FKF1* and *GI* ortholog genes in the soybean genome. (A) The gene structures of *GmFKF1* and *GmFKF2*, compared with that of *AtFKF1* (At1g68050.1). (B) The gene structures of *GmGI1α*, *GmGI1β*, *GmGI2*, and *GmGI3*, compared with that of *AtGI* (At1g22770.1). (C) A phylogenetic tree of the FKF1 proteins from soybean and other plant species. The protein sequences accessions used were AcFKF1 (*Allium cepa*, *GQ232754*), ZmFKF1 (*Zea mays*, *GRMZM2G107945*), ZmFKF2 (*Zea mays*, *GRMZM2G106363*), SbFKF1 (*Sorghum bicolor*, *Sb05g021030*), OsFKF1 (*Oryza sativa*, *Os11g34460*), BdFKF1 (*Brachypodium distachyon*, *Bradi4g16630*), TaFKF1(*Triticum aestivum*, *ABL11478.1*), HaFKF1 (*Helianthus annuus*, *ADO61006.1*), AtFKF1 (*Arabidopsis thaliana*, *At1g68050.1*), MtFKF1 (*Medicago truncatula*, *Medtr4g156890*), GmFKF1 (*Glycine max*, *Glyma05g34530*), GmFKF2 (*Glycine max*, *Glyma08g05130*), McFKF1 (*Mesembryanthemum crystallinum*, *AAQ73528.1*), HvFKF1 (*Hordeum vulgare*, *ACR15149.1*). (D) The phylogenetic relationships among GI homologs. Accession numbers: AcGI (*Allium cepa*, *GQ232756*), OsGI (*Oryza sativa*, *Os01g0182600*), SbGI (*Sorghum bicolor*, *Sb03g003650*), ZmGI (*Zea mays*, *ABZ81992.1*), ZmGI1A (*Zea mays*, *DAA06172.1*), LpGI (*Lolium perenne*, *CAY26028.1*), TaGI1 (*Triticum aestivum*, *AAQ11738.1*), TaGI2 (*Triticum aestivum*, *AAT79486.1*), TaGI3 (*Triticum aestivum*, *AAT79487.1)*, HvGI (*Hordeum vulgare*, *AAW66945.1*), ScGI (*Secale cereal*, *ADR51711.1*), BdGI (*Brachypodium distachyon*, *DV476579*), LgGI (*Lemna gibba*, *BAD97869.1*), MtGI (*Medicago truncatula*, *XP_003592048.1*), PsGI (*Pisum sativum*, *ABP81863.1*), GmGI1α (*Glycine max*, *Glyma20g30980*), GmGI1β (*Glycine max*, *Glyma20g30980*), GmGI2 (*Glycine max*, *Glyma09g07240*), GmGI3 (*Glycine max*, *Glyma10g36600*), PtGI (*Populus trichocarpa*, *XP_002300901.1*), AtGI (*Arabidopsis thaliana*, *At1g22770.1*). (C) and (D) phylogenetic trees were constructed with MEGA 4.0 software. Full-length amino acid sequences were aligned and Bootstrap analysis was performed based on 1,000 replicates.

The coding sequences of *FKF1* and *GI* genes homologs in soybean genome were respectively isolated by BLAST search against the soybean database (http://www.phytozome.org) using *Arabidopsis FKF1* and *GI* sequences as the query, and then cloned from the soybean cultivar Kennong 18 (Table S1 in File S1). The soybean genome contained two *FKF1* homologs (hereafter *GmFKF1* and *GmFKF2*, *Gm* for *Glycine max*), and three *GI* homologs (hereafter *GmGI1*, *GmGI2*, and *GmGI3*). Interestingly, alternative splicing occurred in the 11^th^ exon of *GmGI1*, resulting in two different versions (namely *GmGI1a* and *GmGI1β*). In the same way, one of *CYCLING DOF FACTOR 1* (*CDF1*) homologs (namely *GmCDF1*) was also obtained. *FKF1* and *GI* homologs in soybean shared comparable exon sizes and similar gene structures with those in *Arabidopsis*, but the intron size of soybean genes were considerably larger (Figure 1A, B), indicating more complex regulation for soybean *FKF1* and *GI* homologs. GmFKF1 and GmFKF2 shared 95% of amino acids identity with each other. Both GmFKF1 and GmFKF2 shared high peptide identities with AtFKF1 (83.5% and 81.4%, respectively), and included all the known functional domains in AtFKF1: the LOV domain, F-box motif and Kelch repeats (Figure S1 in File S1). The LOV domains from GmFKF1 and GmFKF2 shared high similarity with that from AtFKF1, AtPHOT1 and AtPHOT2 in the secondary structure elements [69] (Figure S2 in File S1). Similarly, the protein sequences of GmGI1a, GmGI1β, GmGI2, and GmGI3 were highly conserved with approximately 76% of amino acids identities with AtGI. GmGI1 shared peptide identity of 94%∼97% with GmGI3, and 80%∼82% with GmGI2. The GmGIs proteins also contained multiple transmembrane domains and nuclear localization signals in similar positions as in the GIs from *Arabidopsis*, *Triticum* and *Brachypodium* (Figure S3 in File S1).

To examine the evolutionary relationships of *FKF1* or *GI* homologs from several plant species, phylogenetic trees were constructed using MEGA4.0 software (Figure 1C, D). Both trees clearly divided into two major clades, one corresponding to the monocots, and the other to the dicots. The GmFKFs and GmGIs proteins were clustered within the latter clade, and within the legume sub-clade together with FKF1 and GI homologs from *Medicago truncatula* and *Pisum sativum*. Interestingly, GmGI1 and GmGI3 were more closely to the *Medicago truncatula* and *Pisum sativum* GI homologs than to GmGI2, and *GmGI2* had much shorter introns than *GmGI1* and *GmGI3* (Figure 1B), suggesting that *GmGI1* and *GmGI3* diverged from *GmGI2* before soybean speciation.

### Diurnal Rhythms of *FKF1* and *GI* Transcripts in Soybean

**Figure 2:**
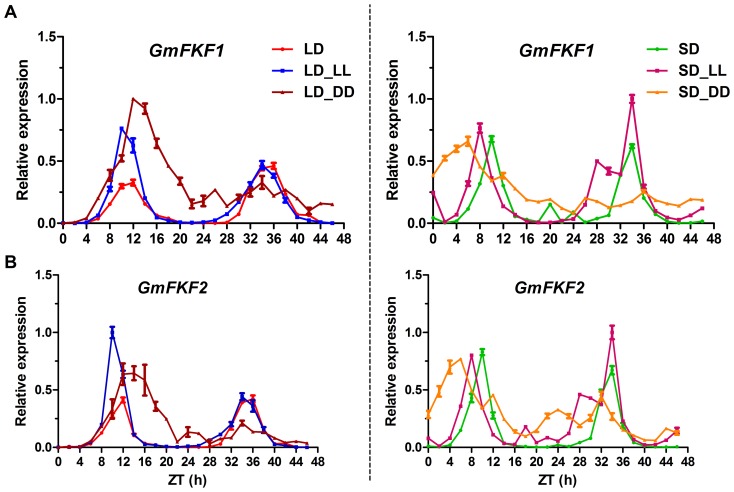
Circadian rhythms of *FKF1* homologs in soybean. *GmFKF1* (A) and *GmFKF2* (B) gene expression under different light regimes. LD, 16 hr light/8 hr dark; SD, 8 hr light/16 hr dark; LL, constant light; DD, constant dark. Soybean gene *ACT11* was used as a control for normalization.

In *Arabidopsis*, the transcription of *FKF1* and *GI* is both controlled by the circadian clock and photoperiod [13]. To determine whether the expression of *GmFKF*s and *GmGI*s fluctuate diurnally, RT-qPCR was performed using unifoliolate leaves under both LD and SD conditions. The level of *GmFKF1* and *GmFKF2* transcripts shared nearly the same patterns under both light regimes and showed clear circadian rhythms, with a peak at Zeitgeber time 12 h (ZT12) in LD and at ZT10 in SD, respectively (Figure 2). Such patterns were consistent with those observed in *Arabidopsis* and *Nicotiana* [10], [26], [70], and different from that of *AcFKF1* (onion *FKF1*), whose transcript accumulation varied greatly according to the photoperiodicity [43].

To determine whether the diurnal rhythms of *GmFKF1* and *GmFKF2* transcripts were stable under free running conditions, the seedlings grown for one week in LD or SD were respectively transferred to continuous light (LL) and continuous dark (DD) conditions. The diurnal rhythms of *GmFKF1* and *GmFKF2* expression levels in LD continued cycling under LL and DD conditions and peaked 2 hr earlier in LD-LL (Figure 2). Differently, the peaking values were significantly higher on the first cycle of LD-LL and LD-DD conditions. Similarly, *GmFKF1* and *GmFKF2* transcripts still maintained circadian rhythms in SD-LL, but showed a longer period of 2 hr than in SD. In SD-DD, *GmFKF1* and *GmFKF2* transcript levels peaked 4 hr in advance on the first cycle, and then the circadian rhythms gradually damped (Figure 2). Summarily, day-length greatly affected the circadian rhythms of *GmFKF1* and *GmFKF2* transcripts and light influenced expression levels.

**Figure 3:**
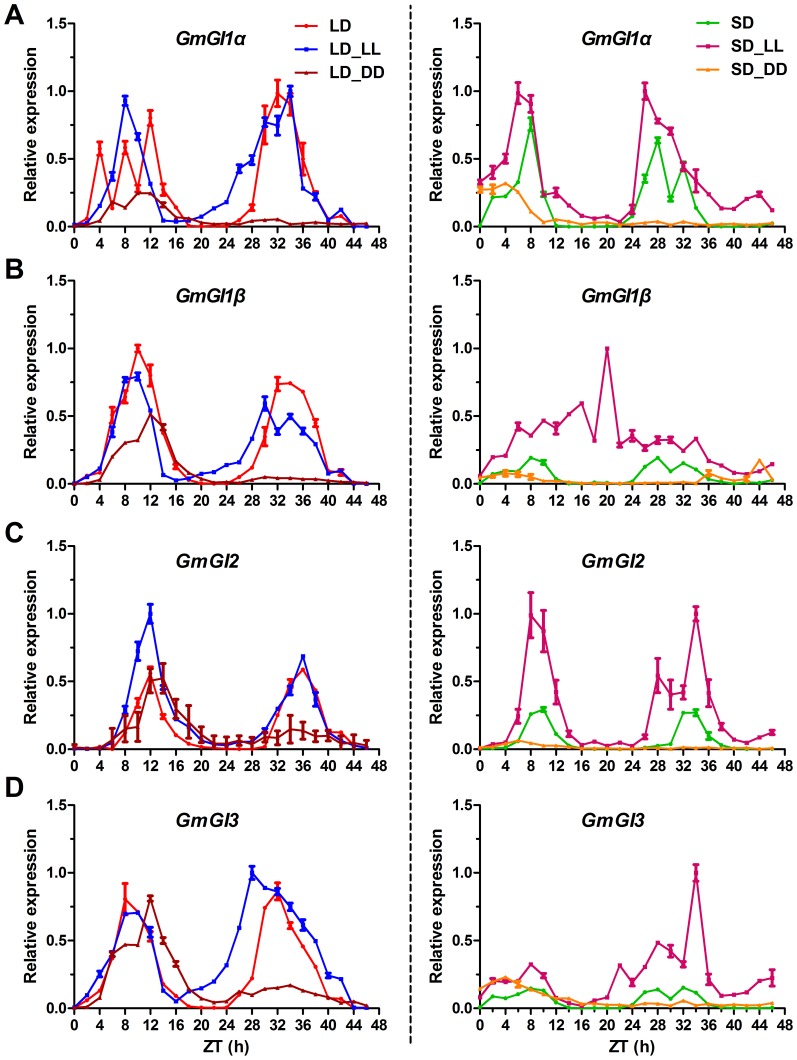
Circadian rhythms of *GmGIs* transcript levels. *GmGI1α* (A), *GmGI1β* (B), *GmGI2* (C) and *GmGI3* (D) gene expression under different light regimes**.** LD, 16 hr light/8 hr dark; SD, 8 hr light/16 hr dark; LL, constant light; DD, constant dark. Soybean gene *ACT11* was used as a control for normalization.

In *Arabidopsis*, the circadian rhythms of *FKF1* and *GI* transcript levels were in phase in LD [26], [27]. Therefore, the diurnal expression patterns of *GmGIs* were also conducted in the same way. *GmGI2* transcripts showed clear circadian rhythms under LD and LD-LL as well as LD-DD conditions, and peaked at ZT12 (Figure 3C). The rhythmic phase of *GmGI2* transcripts in SD and SD-LL resembled that of *GmFKF1* and *GmFKF2* transcripts. Compared with *GmFKF1* and *GmFKF2*, *GmGI2* expression levels were greatly affected by light, much higher in SD-LL and much lower in SD-DD than in SD (Figure 3C). The circadian rhythms of *GmGI1* and *GmGI3* transcripts were weaker or much irregular under both LD-entrained and SD-entrained conditions compared with that of *GmGI2*. In LD, *GmGI1* transcripts had high levels at ZT10∼12, while the highest level of *GmGI3* transcripts appeared at ZT8. The circadian rhythms of *GmGI1* and *GmGI3* transcripts continued cycling in LD-LL and LD-DD, but the cycling periods were longer or shorter than 24 hr (Figure 3A, B, D). In SD, *GmGI1* and *GmGI3* transcripts showed much lower levels than in LD, but the diurnal rhythms still oscillated with a period of about 24 hr. In SD-LL, *GmGI1* and *GmGI3* transcript levels apparently increased. Of these, *GmGI1α* showed diurnal rhythm and a shorter period, while *GmGI1β* and *GmGI3* hardly showed circadian rhythms. In SD-DD, the expression levels of *GmGI1* and *GmGI3* reduced to nearly be undetectable (Figure 3A, B, D). Thus, the circadian expressions of *GmFKF*s and *GmGI*s were diurnally regulated, and the expression patterns of *GmGI2* and *GmFKF*s were in phase under both LD and SD conditions **(** Figure 2, 3 **)**. Although GmGI1 and GmGI2 as well as GmGI3 shared high peptide identity each other, the diurnal expression patterns of them apparently differed.

### Spatial and Temporal Expression Profiles of *GmFKF*s and *GmGI*s

**Figure 4:**
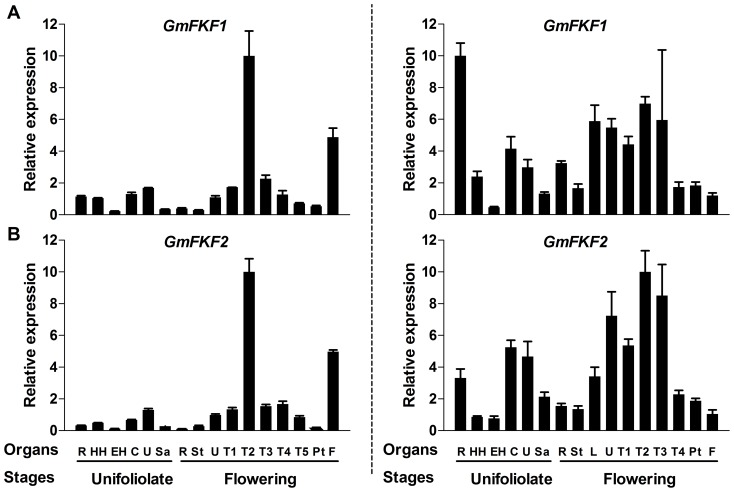
Expression profiles of *GmFKFs* in various tissues/organs. *GmFKF1* (A) and *GmFKF2* (B) expression levels were investigated in both LD (left panel) and SD (right panel). Samples were collected at ZT12 in LD (left panel) and at ZT8 in SD (right panel). The soybean *UKN1* gene was used as the normalization transcripts and Bars indicate standard deviation. R: roots; HH: hypocotyls; EH: epicotyls; C: cotyledons; U: unifoliolates; Sa: shoot apex meristem; St: stems; L: lateral leaves; T1: fully opened 1^st^ Trifoliolates; T2: fully opened 2nd Trifoliolates; T3: fully opened 3rd Trifoliolates; T4: fully opened 4th Trifoliolates; T5: fully opened 5th Trifoliolates; Pt: petioles; F: flowers.

**Figure 5:**
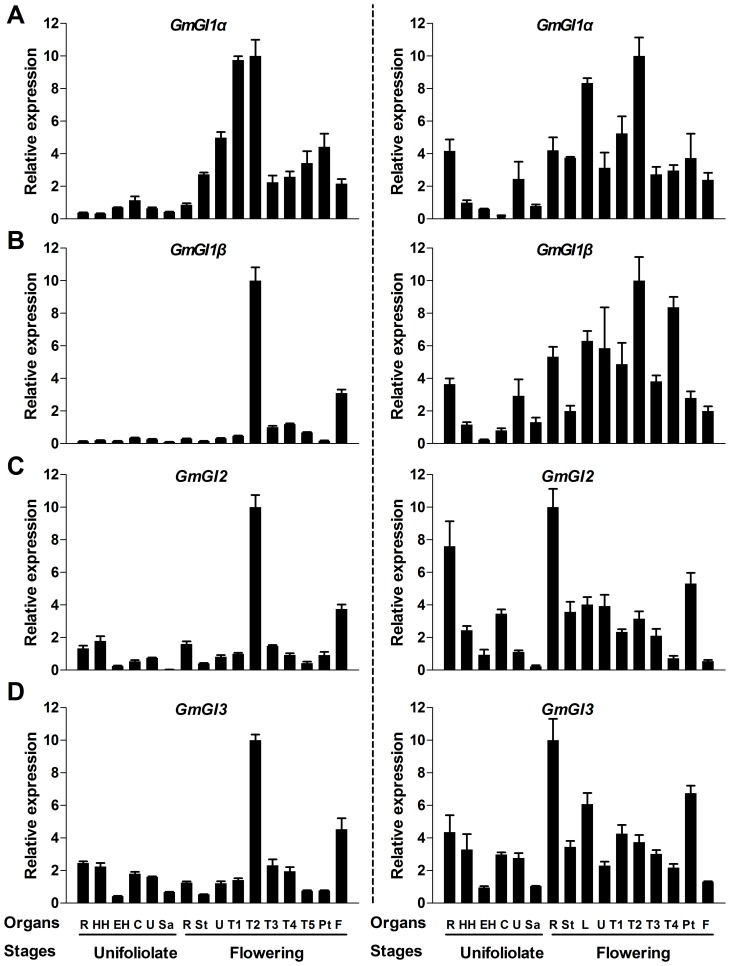
Expression profiles of *GmGIs* in various tissues/organs. The expression of *GmGI1α* (A), *GmGI1β* (B), *GmGI2* (C) and *GmGI3* (D) were investigated in LD (left panel) and SD (right panel). The samples were collected as Figure 4. The soybean *UKN1* gene was used as the reference gene.

To better understand *GmFKF* **s** and *GmGI* **s** functions, spatio-temporal expression patterns of *GmFKF* **s** and *GmGI* **s** were systematically performed using RT-qPCR. *GmFKF* **s** and *GmGI* **s** shared similarly tissue/organ-specific expression patterns and were detected in a variety of tissues/organs under both light regimes. Moreover, *GmFKF* **s** and *GmGI* **s** showed higher expression levels in most organs in SD than in LD (Figure 4, 5). Under LD conditions, *GmFKF* and *GmGI* transcripts had the highest level in the 2nd trifoliolates and floral buds at flowering. Under SD conditions, *GmFKF*s and *GmGI1* showed the highest expression levels in roots at unifoliolate opening and in leaves at flowering (Figure 4, 5A, B). However, *GmGI2* and *GmGI3* had the highest level in roots at both vegetative and reproductive phases (Figure 5C, D). It has been reported that *AtFKF1* mRNA was detected throughout the plant, with the highest level in leaves [10] and that *AtGI* had higher expression level in the inflorescence apices, young flowers, and young siliques [27]. Therefore, *GmFKF* **s** and *GmGI* **s** displayed different tissue/organ expression patterns as day-length changed.

**Figure 6:**
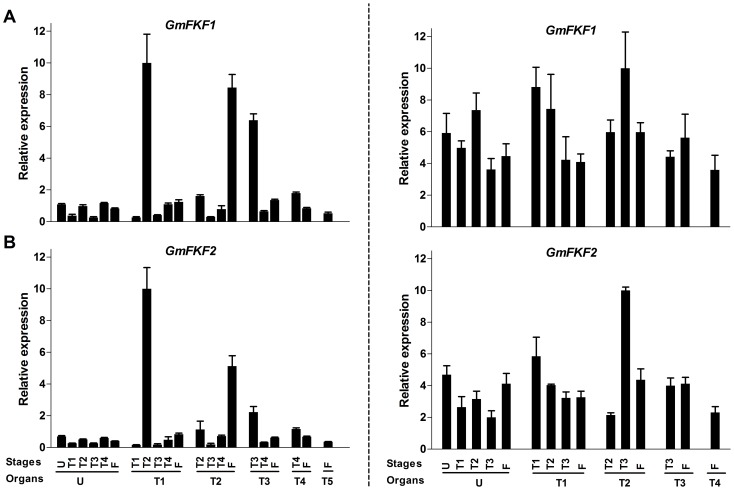
Expression profiles of *GmFKFs* during development. *GmFKF1* (A) and *GmFKF2* (B) gene expression were performed in leaves. The samples were collected in both LD (left panel) and SD (right panel). The developmental stages included U (fully opened unifoliolates) stage, T1 (fully opened 1^st^ Trifoliolates) stage, T2 (fully opened 2nd Trifoliolates) stage, T3 (fully opened 3rd Trifoliolates) stage, T4 (fully opened 4th Trifoliolates) stage and T5/F (fully opened 5th Trifoliolates) stage. The gene *GmUKN1* was used as a control and bars indicated standard deviation.

**Figure 7:**
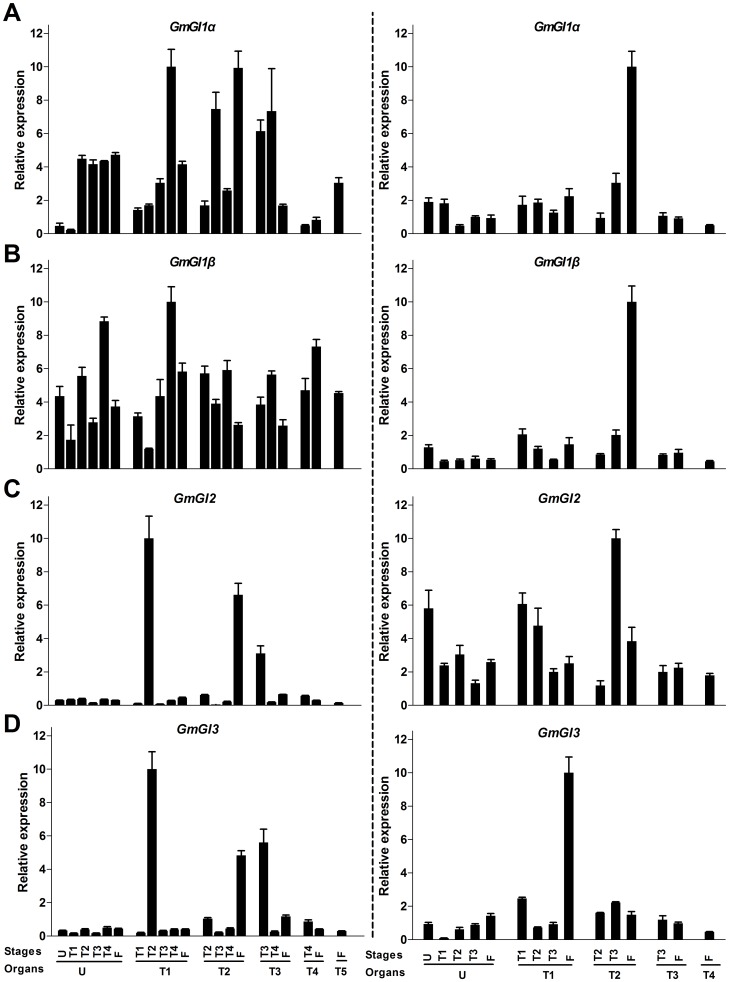
Expression profiles of *GmGIs* during development. *GmGI1α* (A), *GmGI1β* (B), *GmGI2* (C) and *GmGI3* (D) expression levels were performed in leaves. The samples and developmental stages in LD (left panel) and SD (right panel) were the same as Figure 6. The soybean *UKN1* gene was used as the normalization transcripts.

The expression patterns of *GmFKF* **s** and *GmGI* **s** during the developmental progress were also carried out. In this case, *GmFKF*s and *GmGI2* shared similar expression patterns in both LD and SD, with very low expression levels in most of leaves in LD. Short days increased *GmFKF* **s** and *GmGI2* expression levels in all leaves and the levels slightly decreased over time (Figure 6, 7C). Contrary to *GmGI2*, *GmGI1* had high transcript levels in LD and low levels in SD at all development stages (Figure 7A, B). The transcript level of *GmGI3* was very low in most of leaves regardless of day-length (Figure 7D). So *GmGI* **s** had totally different day-length responses in their developmental expression patterns. In *Arabidopsis*, *FKF1* and *GI* expression levels increase slightly over time during development, consistent with their role in floral promotion [10], [27]. The results suggested *GmGI2* and *GmFKF* **s** may involve flowering regulation under SD conditions.

### Nuclear Localization of the GmFKF and GmGI Proteins

**Figure 8:**
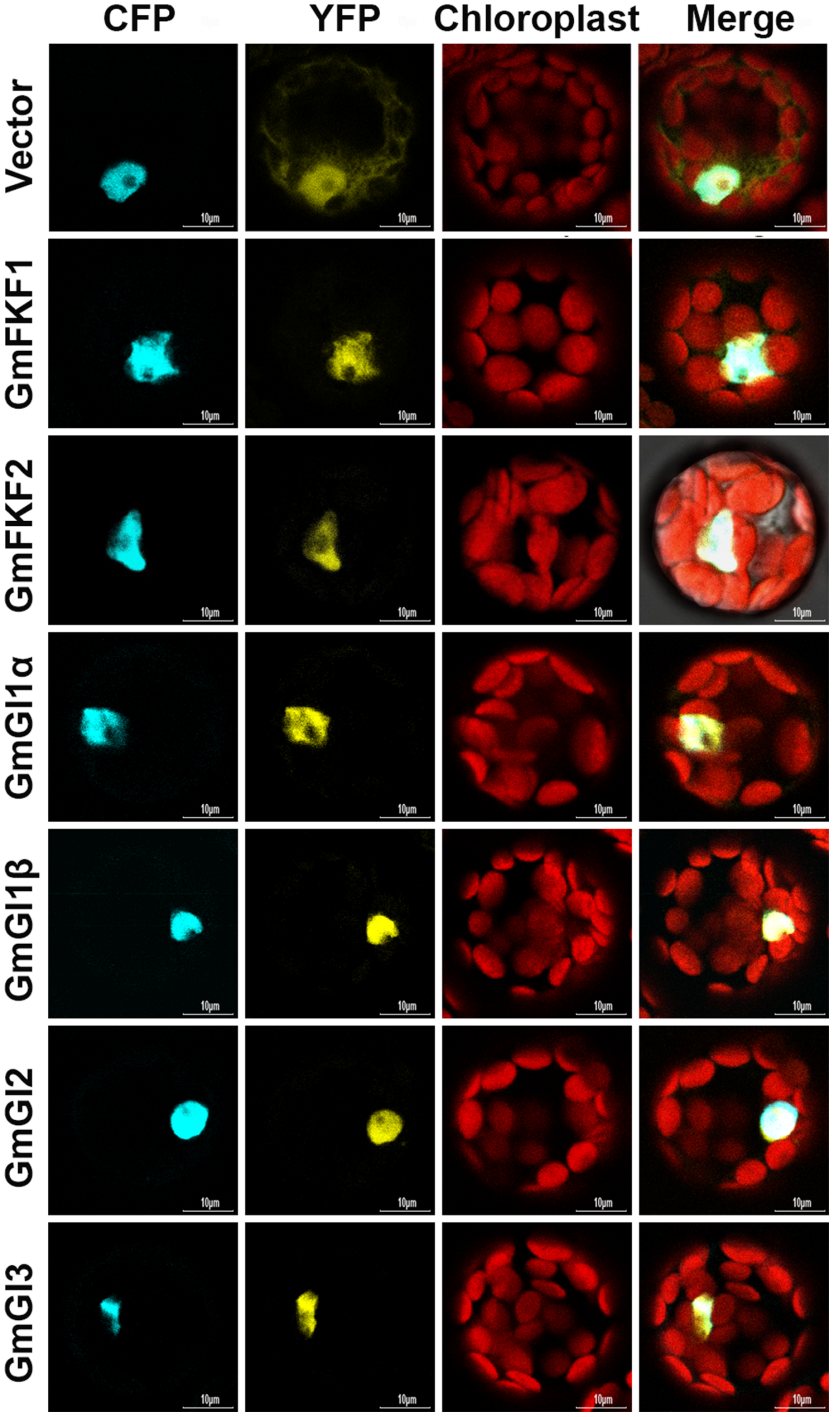
Nuclear localization of GmFKF1, GmFKF2, GmGI1α, GmGI1β, GmGI2 and GmGI3 proteins in *Arabidopsis* protoplasts. The vector indicated the empty vector as negative control; The CFP was for CFP-AHL22, a nuclear marker (Xiao *et al*., 2009); the YFP was for YFP fluorescence; the red signal was due to auto-fluorescence of chloroplasts; the last panel showed superimposition of the former three panels.

To investigate the subcellular localizations of the FKF1 and GI proteins, a yellow fluorescent protein (YFP)-coding sequence was fused to the C-terminus of *GmFKF* and *GmGI* genes driven by a 35S promoter. The fusion constructs of *GmFKF1*/*2*-YFP or *GmGI1α*/*1β*/*2*/*3*-YFP was co-transformed with the nuclear marker gene CFP-*AHL22*, a positive control [71], into *Arabidopsis* mesophyll protoplasts. The results showed that both FKF1 and GI proteins were mainly targeted to the nucleus (Figure 8), similar to the localization patterns of AtFKF1 and AtGI [72], [73]. The nuclear localization of GmGI proteins were consistent with the presence of NLS-like (nuclear localization signals) sequences in the middle of GmGI proteins (Figure S3 in File S1), which were also present in GI homolog proteins in other plants [47], [50], [73].

### Interactions between GmFKF and GmGI Proteins

**Figure 9:**
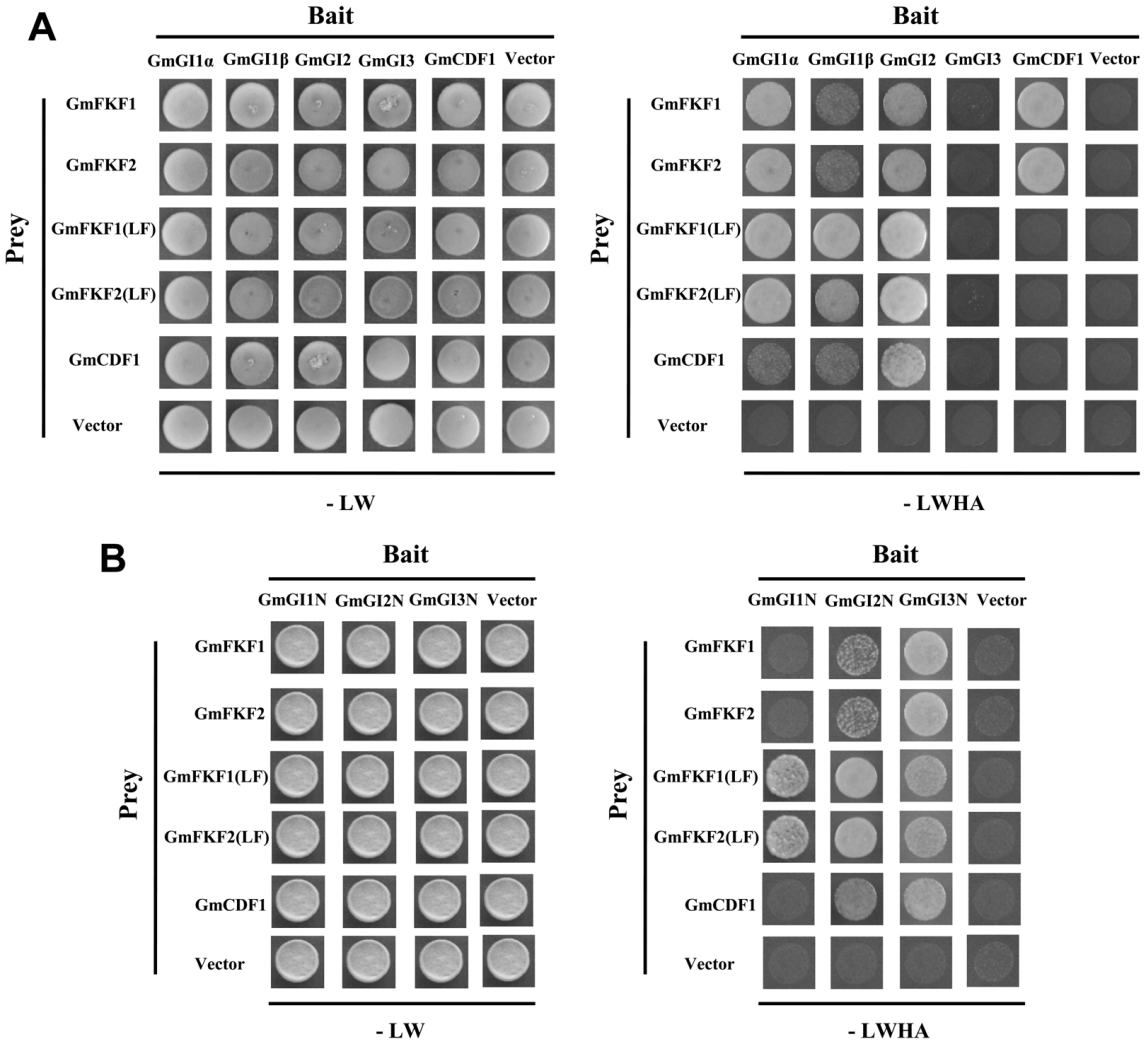
Interactions between GmFKFs and GmGI in yeast. (A) GmFKFs interacted with GmGIs and GmCDF1. GmFKFs (LF) included the LOV domain and F-box motif of GmFKF1 and GmFKF2. (B) GmGIs N interacted with GmFKFs (LF) and GmCDF1. GmGIs N represented the N terminus of GmGIs. -LW, synthetic dropout (SD) yeast growth medium lacking leucine and tryptophan; -LWHA, SD medium lacking Leu, Trp, histidine, and adenine.

**Figure 10:**
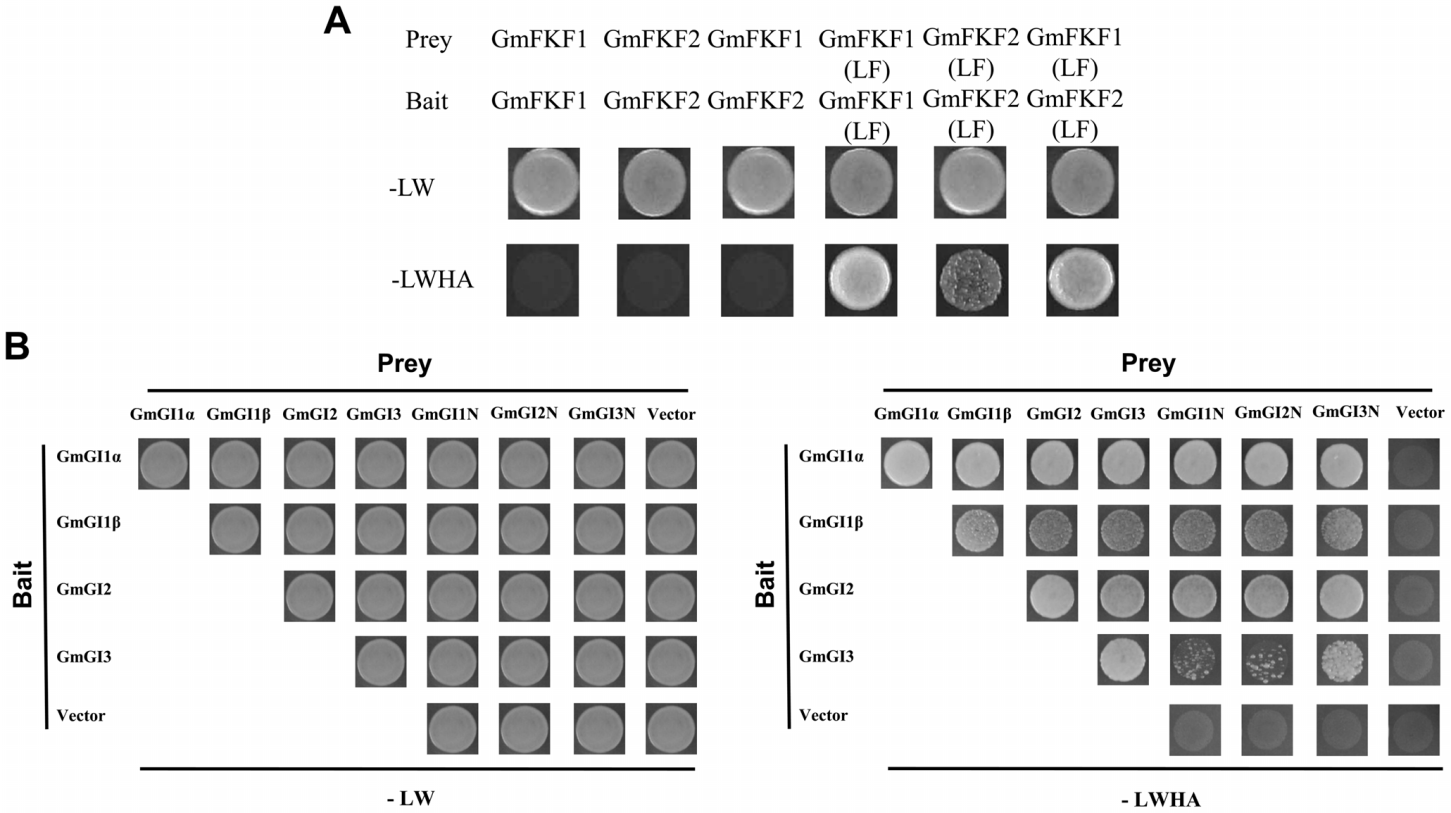
Interactions among GmFKFs(LF) or GmGIs in yeast. (A) Interactions between GmFKF1(LF) and GmFKF2(LF). (B) Interactions among GmGI1, GmGI2 and GmGI3 proteins. “-LW” and “–LWHA” were referred as Figure 9.

In *Arabidopsis*, FKF1 interacts with GI in a blue light dependent manner under LD conditions, and subsequently degrades the *CO* repressor CDF1 and induces *CO* expression [13], [14]. To explore the possible roles of GmFKFs and GmGIs, a series of yeast two-hybrid assays were performed. Both GmFKF1 and GmFKF2 interacted with GmGI1α and GmGI2 in yeast, and the LOV domains from GmFKF1 and GmFKF2 proteins were sufficient to interact with GmGI1α and GmGI2 (Figure 9A), similar to the AtFKF1 LOV domain [13]. Meanwhile, GmFKF1 and GmFKF2 had an interaction with GmCDF1, but no interactions were observed between GmFKF1/GmFKF2 LOV domain and GmCDF1, suggesting the importance of C-terminus Kelch repeats for this interaction (Figure 9A) [14]. In addition, GmGI2 also interacted with GmCDF1 (Figure 9A). Contrary to the AtGI N-terminus [13], the N-terminus of GmGI1 and GmGI2 interacted with GmFKF1/GmFKF2 LOV domain but not the full length of GmFKF1 and GmFKF2 (Figure 9B). Interestingly, GmGI3 N-terminus, not GmGI3 full protein, interacted with full proteins as well as LOV domains of GmFKF1 and GmFKF2 (Figure 9A, B). GmGI2 and GmGI3 N termini also interacted with GmCDF1 (Figure 9B). As expected, the C-terminus of GmGIs did not interact with GmFKF and GmFKF LOV domains (Figure S4 in File S1). It has been reported the phototropin LOV1 form a stable homo-dimer *in vitro* regardless of light conditions [69], so did the GmFKF1/GmFKF2 LOV domains in our test (Figure 10A). Moreover, the interaction was also occurred between the GmFKF1 LOV domain and the GmFKF2 LOV domain (Figure 10A). In *Arabidopsis*, AtGI forms a tetramer in solution [74]. Similarly, the homo- or hetero-dimer were also observed among GmGIs and the N terminus played important roles in these interactions (Figure 10B).

### Potential Roles of *GmFKF1* and *GmFKF2* in Flowering Regulation

**Figure 11:**
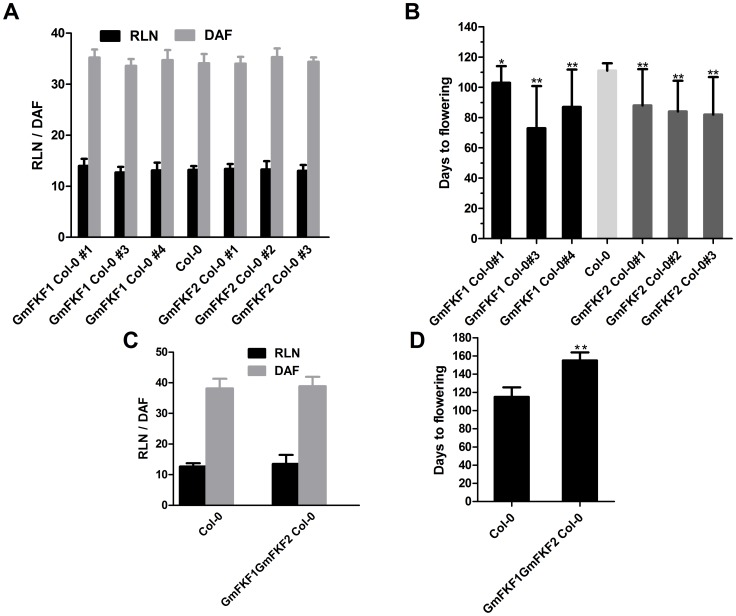
Overexpression of *GmFKF1* or *GmFKF2* affected flowering in transgenic Arabidopsis. Rosette leaf number (RLN) and days to flowering (DAF) under LD (A, C) and SD (B, D) conditions. The single transgenic lines were T2 generation and the double transgenic plants were T1 generation. Error bars denoted the standard deviation. n = 20∼30 plants.

The functions of *GmFKF1* and *GmFKF2* in the regulation of flowering were carried out by overexpressing these genes in *Arabidopsis*. *GmFKF1* and *GmFKF2* were respectively over-expressed in *Arabidopsis* Col-0 background and the transgenic lines (T2 generation) were grown under both LD and SD conditions. Neither the flowering time nor the rosette leaf number of the transgenic lines over-expressing *GmFKF1* or *GmFKF2* had significantly difference from that of the control plants in LD (Figure 11A). However, the transgenic plants flowered much earlier than control plants in SD (Figure 11B). The results indicated that *FKF1* may promote flowering, specifically under SD conditions.

Most of genes in soybean are present in multiple copies resulting from three duplication events occurring in the evolutionary course [75]. To determine whether function redundancy were present between *GmFKF1* and *GmFKF2*, the double transgenic plants (T1 generation) were obtained. Again, there was little difference with respect to flowering time or rosette leaf number between the double transgenic plants and the wild type in LD (Figure 11C). Interestingly, the double transgenic plants flowered approximately one month later than wild type plants in SD (Figure 11D). The results suggested that the functions of *GmFKF1* or *GmFKF2* on flowering promotion in SD were antagonistic each other.

## Discussion

The homolog sequences of *FKF1* and *GI* in soybean have been reported [44], [56], [68], but the biochemical features and molecular functions of FKF1 and GI proteins has not systematically studied. In this study, we obtained the soybean *FKF1* and *GI* homologs by homology-based cloning method. Phylogenetic analysis showed high amino acid sequence identities presented between GmFKFs and AtFKF1 [10], [26] and between GmGIs and other GI proteins [10], [26], [45], [47], [49], [69], [73] (Figure S1 in File S1, and Figure S3 in File S1). In addition, *GmGI2* probably evolved earlier than *GmGI1* and *GmGI3* (Figure 1D). The similar nuclear localizations of GmFKF and GmGI proteins to their homologs in other plants indicated their conserved functions [13], [72].

The expression of *GmFKFs* and *GmGI2* were regulated by both the clock and light (Figure 2, 3C), as that of *FKF1* and *GI* in *Arabidopsis* and other plant species [10], [27], [33], [43], [46], [47], [49]–[51]. However, expression divergence among *GmGI*s was obvious. Compared with *GmGI2*, the circadian rhythms of *GmGI1* and *GmGI3* transcripts were greatly affected by day-length and long days largely contributed to the rhythmic phases of *GmGI1* and *GmGI3* (Figure 3A, B, D). Secondly, day-length also influenced spatio-temporal expression patterns of *GmFKF*s and *GmGI*s. The higher levels of *GmFKF* and *GmGI* transcripts occurred in the second trifoliolates and the floral meristem in LD, but in roots in SD (Figure 4, 5). Contrast to *GmGI2*, the level of *GmGI1* transcripts was much higher in LD than in SD, and *GmGI3* lowly expressed under both LD and SD conditions (Figure 7). Therefore, developmental cues had effects on the expression of *GmGI*s and *GmFKF*s (Figure 6, 7). However, different from *AtFKF1* and *AtGI*, which increase their expression along with development [10], [27], *GmFKF*s and *GmGI2* expression levels reduced slightly in SD over time (Figure 6, 7C).

The synchronous expression of *GmFKF*s and *GmGI*s was conserved and obvious, which displayed in the same time point (circadian rhythms and peaks), the same location (leaves), both vegetative and reproductive stages. And the same subcellular localization (the nucleus) implied interactions probably occurred between GmFKFs and GmGIs. Such interactions were confirmed by yeast two hybrid assays and independent of light (Figure 9, 10), while the FKF1-GI complex formation in *Arabidopsis* is in a blue light dependent manner [13]. The subfunctionalization of *GmFKF*s and *GmGI*s also happened on interactions between different copies of these genes. For example, *GmGI1β* and *GmGI3* transcripts peaked earlier than *GmFKF1* and *GmFKF2* in both light regimes. Consistent with this, neither GmFKF1 nor GmFKF2 interacted with GmGI1β and GmGI3, but did with GmGI1α and GmGI2. In addition, both GmFKF1 and GmFKF2 interacted with GmCDF1, so did GmGI2 (Figure 9A). The LOV domain of GmFKFs may be important for intermediating the interaction with their partners, supported by data from *Arabidopsis* FKF1 [13], [14], [26], [76]–[78]. In soybean, GmFKF1/GmFKF2 LOV domains interacted with GmGI1α and GmGI2 (Figure 9A). Meanwhile, C-terminus Kelch repeats of GmFKFs may be related to an interaction with GmCDF1 (Figure 9A). In *Arabidopsis*, sufficient levels of the FKF1–GI complex in LD are required for the proper induction of *CO* [13], [26], [27]. Therefore, the interactions between GmFKFs and GmGIs may confer to the promotion of flowering in soybean.

In *Arabidopsis*, the *fkf1* mutant flowers much later than wild type in long days, whereas it flowers much normally in short days [10]. On the contrary, both *GmFKF1* and *GmFKF2* promoted soybean flowering in SD, not in LD (Figure 11A, B). However, the promotion effect of *GmFKF1* or *GmFKF2* was inhibited when *GmFKF1* and *GmFKF2* were co-overexpressed (Figure 11D), suggesting *GmFKF1* and *GmFKF2* antagonized each other on the regulation of flowering. Provided that *GmFKF1* and *GmFKF2* shared the synchronous expression, the fine tune between *GmFKF1* and *GmFKF2* was important for soybean flowering regulation. However, overexpression of *GmGI*s had no significant difference in flowering time and *35S::GmGI1α* and *35S::GmGI1β* could not rescue *gi-1* mutant phenotype (data not shown), inferring soybean *GmGI*s had a species-specific flowering activity and the *FKF1-GI–CDF1–CO*-*FT* pathway was unique in soybean. Additionally, the transgenic plants over-expressing both *GmFKF2* and *GmGI1α* produced more rosette leaves and exhibited more vigorous growth and senescence retardation compared to the wild type plants in LD (Figure S5 in File S1), suggesting their functions on vegetative growth. *GmGI3*, which may be a more ancestral gene than *GmGI1* and *GmGI2*, may had a subtle function in flowering regulation, because the full protein did not interact with GmFKF1/2, but its N-terminus did interact with GmFKF1/2 and GmCDF1 (Figure 9). Consistent with this, it has been reported the truncated protein (735aa) of GmGIa (corresponding to GmGI3 in this study) showed a significantly earlier flowering phenotype than the wild type under natural day length conditions [56]. Undoubtedly, more information *in situ* not *ex situ* regarding the biochemical functions of the soybean *FKF1* and *GI* genes and their mutants in soybean needed to elucidate their *bona fide* functions.

## Materials and Methods

### Plant Materials and Growth Conditions

Ectopic expressing materials were *Arabidopsis thaliana* wild-type Col-0 stored in our lab. Seeds were pretreated at 4°C for 3 days in the dark after treated with sodium hypochlorite, then transferred to a growth chamber 22°C under long days (LD, 16 h light/8 h dark) or short days (SD, 8 h light/16 h dark). Light intensity was approximately 100 µmol·m^−2^·s^−1^ provided by white fluorescent illumination. Kennong 18 (KN18), a soybean cultivar, was used in this study. Seedlings were grown in the phytotron under long-day (LD, 16 h light/8 h dark) or short-day (SD, 8 h light/16 h dark) conditions. The temperature was 26°C. The light source was from cool-white fluorescent illumination. The samples used for analysis of tissue-organ expression patterns were harvested as described by Hu et al [79]. The samples during development (from fully expanded unifoliolate stage until to flowering onset) comprised unifoliolate leaves and different trifoliolate leaves as well as flower buds. The samples for spatio-temporal expression analysis were harvested at ZT12 in LD and at ZT8 in SD. The fully expanded unifoliolate leaves were harvested as photoperiod samples at 2 hr intervals over 48 hr when seedlings were exposed to either long-day or short-day conditions, then the seedlings were transferred to either constant light (LL) or constant darkness (DD) conditions and the samples were continuously collected as in LD or SD. All samples were immediately frozen in liquid nitrogen and stored at −80°C until required.

### Isolation and Phylogenetic Analysis of Soybean *FKF1* and *GI* Homologs

The *Arabidopsis* FKF1 and GI amino acid sequences have been used as baiters to search for potential homologous genes in the soybean database (Phytozome 4.0), and homologs sequences were acquired based on the E value  = 0 and then used to conduct BLASTP in Arabidopsis database (http://www.arabidopsis.org/Blast/index.jsp) for preliminary screening. Finally, two *FKF1* and three *GI* homologs were determined based on further analysis in Softberry database (http://www.softberry.com/berry.phtml). Gene-specific primers were designed at both ends of gene non-coding/coding regions and used to conduct nested or semi-nested PCR employed KN18 cDNA as the template. All primers used were listed in Table S2 in File S1. The PCR products were introduced into the pEASY-T1 vector (TransGen Biotech, CHN) after purification and several independent clones were sequenced for real sequences.

Phylogenetic analysis of FKF1 and GI homologs were conducted, respectively. Predicted and published protein sequences were obtained from NCBI (http://blast.ncbi.nlm.nih.gov/Blast.cgi) and then selected in Pfam database based on functional domains annotation. Multiple alignments were conducted using ClustalX. Phylogenetic neighbor-joining trees were constructed with MEGA 4.0 based on the full-length amino acid sequences. Bootstrap analysis was performed estimate nodal support based on 1,000 replicates.

### RNA Isolation, cDNA Preparation and Real-time Quantitative RT-PCR

Total RNA was extracted using TRIzol reagent (Invitrogen, CA, USA) according to the manufacturer’s recommendations. Contaminating genomic DNA was removed with the RNA-free DNAseI kit (Invitrogen, CA, USA). RNA purity was controlled by determining the ratio of A260/A280 ranging between 1.8 and 2.0 and A260/A230 greater than 2.0. First-strand cDNA was synthesized using 5 µg purified RNA with the M-MLV reverse transcriptase kit (Invitrogen, CA, USA) and oligo-dT primers, according to the manufacturer's protocol. Then the cDNA product was diluted 1∶20 prior to use.

RT-qPCR reaction was performed as previously described [79]. Each reaction was performed in three technical replicates and the Ct value was exported using the StepOne Software v2.0 (ABI Applied). The blank controls were included with H_2_O as the template for each reaction. The soybean *UKN1* gene was used as reference control for tissue-organ and developmental genes analysis, while the gene *ACT11* was used as the normalization transcripts for diurnal rhythms analysis [79]. Relative expression levels were calculated according to the formula: the value = 2^–ΔΔCT^. All primers used were listed in Table S2 in File S1.

### Transient Expression in *Arabidopsis* Protoplasts

The coding sequences (CDS) of *GmFKF1*/*GmFKF2*/*GmGI2* were cloned into pEXSG-YFP by gateway approach. Similarly, the CDS of *GmGI1α*/*GmGI1β*/*GmGI3* were cloned into the vector pENSG–YFP. All of the fusion constructs were driven by the cauliflower mosaic virus 35S promoter. The vector pENSG-CFP-*AHL22*, a nuclear marker gene [71] was co-transformed with each target gene. Meanwhile, the vector pEXSG-YFP was co-transformed with pENSG-CFP-*AHL22* served as negative control. Finally, the *Arabidopsis* protoplasts were visualized by confocal laser scanning microscope (Leica, USA). The protoplasts extraction and transformation were conducted according to the *Arabidopsis* protoplast extraction and plasmid transformation protocols [80].

### Yeast Two Hybrid Assay

Protein–protein interaction was performed using the yeast two-hybrid system according to the Clontech Yeast Protocols PT3024-1. The bait vector pGBKT7-DEST and the prey vector pGADT7-DEST were both Gateway-compatible vectors provided by Cui lab [81]. The protein-coding sequences of GmFKFs and GmGIs were introduced into pGBKT7-Rec and pGADT7-Rec respectively by using the gateway approach. The truncated proteins of GmFKF1 and GmFKF2 consisting of the LOV domain and F-box motif and the GmGIs N terminus as well as the GmGIs C terminus were also subcloned into pGBKT7-Rec and pGADT7-Rec, respectively. The truncated proteins length of GmGIs N terminus and GmGIs C terminus were determined by alignment of amino acid sequences between GmGIs and AtGI [13]. The bait and prey pairs were co-transformed into the yeast strain AH109 (*MATa*, *trp1*, *leu2*) and the positive colonies were screened by growing on the SD dropout medium (-Trp/−Leu/−His/−Ade). Negative controls and self-activation experiments were also investigated. Transformation was conducted according to the manufacturer’s protocol (Clontech). The experiment was performed in three biological replicates and reciprocal hybrids were included.

### Ectopic Expression in *Arabidopsis*

The coding sequences of *GmFKFs* and *GmGI1* genes were introduced into the expression vectors pLeela (Basta-resistance) and pGWB2 (Kanamycin and Hygromycin resistance) by using gateway method, then individually or collectively transformed into *Arabidopsis* wild type Col-0 plants using the floral dipping method [82], which were infected with *Agrobacterium tumefaciens* strain pGV3101 MP90RK. The transgenic plants were screened using 50 mg L^−1^ glufosinate ammonium or 50 mg L^−1^ hygromycin or both. At least three independent transgenic lines were used to measure the flowering time and rosette leaf number. The transgenic lines and the control plants were grown under the same conditions in growth chamber.

## Supporting Information

File S1 **Table S1 A list of** ***FKF1*** **s and** ***GI*** **s as well as** ***CDF1*** **homolog genes in soybean. Table S2 A list of primers for gene cloning and RT-qPCR. Figure S1 Amino acid sequence alignment of GmFKFs (** ***Glycine max*** **) and AtFKF1 (** ***Arabidopsis thaliana*** **).** Identical and similar amino acids were indicated in black-shaded and grey-shaded, respectively. The LOV domain, F-box motif, and Kelch repeats were highlighted in yellow fonts, green fonts and pink fonts, respectively. **Figure S2 Comparison of conserved amino acid sites among several LOV domains.** These LOV domains were from *Glycine max* FKF1 and FKF2, *Arabidopsis thaliana* FKF1, PHOT1_LOV1, PHOT1_LOV2, PHOT2_LOV1 and PHOT2_LOV2. The conserved cysteines were highlighted in black background. The loop regions of GmFKF1/GmFKF2/AtFKF1 LOV were in white fonts and green-shaded. The predicted secondary structure elements of α-helices and β-strands were respectively indicated in red fonts and blue fonts. The numbers in the brackets indicated the amino acid positions these LOV domains started and ended. **Figure S3 Alignment of conserved amino acid sites in GI proteins from several species.** These GI proteins included *Glycine max* GI (GmGIs), *Arabidopsis thaliana* GI (AtGI), *Triticum astivum L.* GI (TaGIs) and *Brachypodium distachyon* GI (BdGI). The black and grey regions represented identical and similar amino acids, respectively. The red region represented transmembrane domains and the blue region represented NLS-like (nuclear localization signals) motifs. **Figure S4 Interactions between GmGI C-terminal and GmFKFs or GmFKF (LF) truncated proteins in yeast.** GmFKFs (LF) included the LOV domain and F-box motif of GmFKF1 and GmFKF2. GmGI C-terminal were fused to the GAL4 activation domain (Prey). GmFKFs and GmFKFs (LF) were fused to the GAL4 DNA binding domain (Bait). The empty vector only contained the activation domain (AD). -LW, synthetic dropout (SD) yeast growth medium lacking leucine and tryptophan; -LWHA, SD medium lacking leucine, tryptophan, histidine, and adenine. **Figure S5 Phenotypes of** ***GmGI1α*** **and** ***GmFKF2*** **co** ***-*** **overexpressing lines in Arabidopsis Col under LD conditions.** The double transgenic plants exhibited more vigorous growth and senescence retardation and produced more rosette leaves (A). Comparison of the rosette leaf number (B) and the days to flowering (C) between the double transgenic plants and the control plants.
(DOCX)
