# Supplementary material for: Identification and Molecular Characterization of FKF1 and GI Homologous Genes in Soybean
Source: PLoS One. 2013 Nov 13;8(11):e79036. doi: 10.1371/journal.pone.0079036 (PMC3827303; doi:10.1371/journal.pone.0079036)
Supplement: File S1 — Table S1 A list of FKF1 s and GI s as well as CDF1 homolog genes in soybean. Table S2 A list of primers for gene cloning and RT-qPCR. Figure S1 Amino acid sequence alignment of GmFKFs ( Glycine max ) and AtFKF1 ( Arabidopsis thaliana ). Identical and similar amino acids were indicated in black-shaded and grey-shaded, respectively. The LOV domain, F-box motif, and Kelch repeats were highlighted in yellow fonts, green fonts and pink fonts, respectively. Figure S2 Comparison of conserved amino acid sites among several LOV domains. These LOV domains were from Glycine max FKF1 and FKF2, Arabidopsis thaliana FKF1, PHOT1_LOV1, PHOT1_LOV2, PHOT2_LOV1 and PHOT2_LOV2. The conserved cysteines were highlighted in black background. The loop regions of GmFKF1/GmFKF2/AtFKF1 LOV were in white fonts and green-shaded. The predicted secondary structure elements of α-helices and β-strands were respectively indicated in red fonts and blue fonts. The numbers in the brackets indicated the amino acid positions these LOV domains started and ended. Figure S3 Alignment of conserved amino acid sites in GI proteins from several species. These GI proteins included Glycine max GI (GmGIs), Arabidopsis thaliana GI (AtGI), Triticum astivum L. GI (TaGIs) and Brachypodium distachyon GI (BdGI). The black and grey regions represented identical and similar amino acids, respectively. The red region represented transmembrane domains and the blue region represented NLS-like (nuclear localization signals) motifs. Figure S4 Interactions between GmGI C-terminal and GmFKFs or GmFKF (LF) truncated proteins in yeast. GmFKFs (LF) included the LOV domain and F-box motif of GmFKF1 and GmFKF2. GmGI C-terminal were fused to the GAL4 activation domain (Prey). GmFKFs and GmFKFs (LF) were fused to the GAL4 DNA binding domain (Bait). The empty vector only contained the activation domain (AD). -LW, synthetic dropout (SD) yeast growth medium lacking leucine and tryptophan; -LWHA, SD medium lacking leucine, tryptophan, histi [file pone.0079036.s001.docx]

**Supplemental data**

**Tables:**

Table S1 A list of *FKF1*s and *GI*s as well as *CDF1* homolog genes in soybean.

| **Gene symbol** | **Locus Tag** | **Protein length (aa)** |
| --- | --- | --- |
| *GmFKF1* | *Glyma05g34530.1* | 620 |
| *GmFKF2* | *Glyma08g05130.1* | 632 |
| *GmGI1a* | *Glyma20g30980.1* | 1175 |
| *GmGI1β* | *Glyma20g30980.1* | 990 |
| *GmGI2* | *Glyma09g07240.1* | 1165 |
| *GmGI3* | *Glyma10g36600.1* | 1177 |
| *GmCDF1* | *Glyma04g33410.1* | 335 |

Table S2 A list of primers for gene cloning and RT-qPCR.

| **Gene names** | **Primer names** | **Primer sequences (5’-3’)** | **Fragment size (bp)** | **Annotation** |
| --- | --- | --- | --- | --- |
| *GmFKF1* | *GmFKF1*_ F  *GmFKF1*_R | ATGGCTGTGACAAAAGAGAAAGAGGAT  TCACAGGTCAGAGTCCTGTCGAC | 1863 | Gene cloning |
| *GmFKF2* | *GmFKF2*_ F  *GmFKF2*_R | ATGGCCATGCCAAAAGAGAAA  TCACAGGTCAGAGTCATGTCGA | 1899 | Gene cloning |
| *GmGI1a* | *GmGI1α*_F  *GmGI1α*_R | ATGTCGTCGGCTTCTTCTTTGATGG  TCACATGGAAATAGTACAGCCTAACTC | 3528 | Gene cloning |
| *GmGI1β* | *GmGI1β*_F  *GmGI1β*_R | ATGTCGTCGGCTTCTTCTTTGATGG  TCATGTTAGTGGCAAACGTAATTTATG | 2973 | Gene cloning |
| *GmGI2* | *GmGI2*_F  *GmGI2*_R | ATCAGATCCGTATACAAAACTCCTCAG  GAGAGATGGGATAACAATTTCTAGC | 3553 | Gene cloning |
| *GmGI3* | *GmGI3*_F  *GmGI3*_R | ATCGCCTCTACACACAAGGATTCAG  GCAAACCCGTTAGTTCCATAAGCAT | 3584 | Gene cloning |
| *GmCDF1* | *GmCDF1*_F  *GmCDF1*_R | ATGGACACCAAGTTCTGCTACTACA  TCAAGAGTTCTCATGAAAGTTAAGGG | 1008 | Gene cloning |
| *GmFKF1* | *GmFKF1*_qF  *GmFKF1*_qR | GCATAAAACCAGACCCTCTTCTCTC  CAATCATGTAATAGTGGCTCATATAC | 111 | Real-time PCR |
| *GmFKF2* | *GmFKF2*_qF  *GmFKF2*_qR | AAAGATTTTGGGTGGGTTTCC  GTGTAATAGACGGTGGAGGAG | 146 | Real-time PCR |
| *GmGI1a* | *GmGI1α*_qF  *GmGI1α*_qR | CTCGAGATGCATTTGGTTGTCTTC  CCAATACATCACCAACAATTAATC | 104 | Real-time PCR |
| *GmGI1β* | *GmGI1β*_qF  *GmGI1β*_qR | GGGCTGGAGACAGGTTGTTGATGC  GTTAGTGGCAAACGTAATTTATG | 135 | Real-time PCR |
| *GmGI2* | *GmGI2*_qF  *GmGI2*_qR | GTAAACCTTCTCATTCTGCTAG  CATTGCTTGAAGTCGTGTTTGGG | 95 | Real-time PCR |
| *GmGI3* | *GmGI3*_qF  *GmGI3*_qR | CTGGATAGAACAGACTTGTACAG  GGAAATCCTGCCTGAACATAC | 154 | Real-time PCR |
| *GmACT11* | *GmACT11*_qF  *GmACT11*_qR | ATCTTGACTGAGCGTGGTTATTCC  GCTGGTCCTGGCTGTCTCC | 126 | Reference gene in Real-time PCR |
| *GmUKN1* | *GmUKN1*_qF  *GmUKN1*_qR | TGGTGCTGCCGCTATTTACTG  GGTGGAAGGAACTGCTAACAATC | 74 | Reference gene in Real-time PCR |

**Supplemental figures:**

**
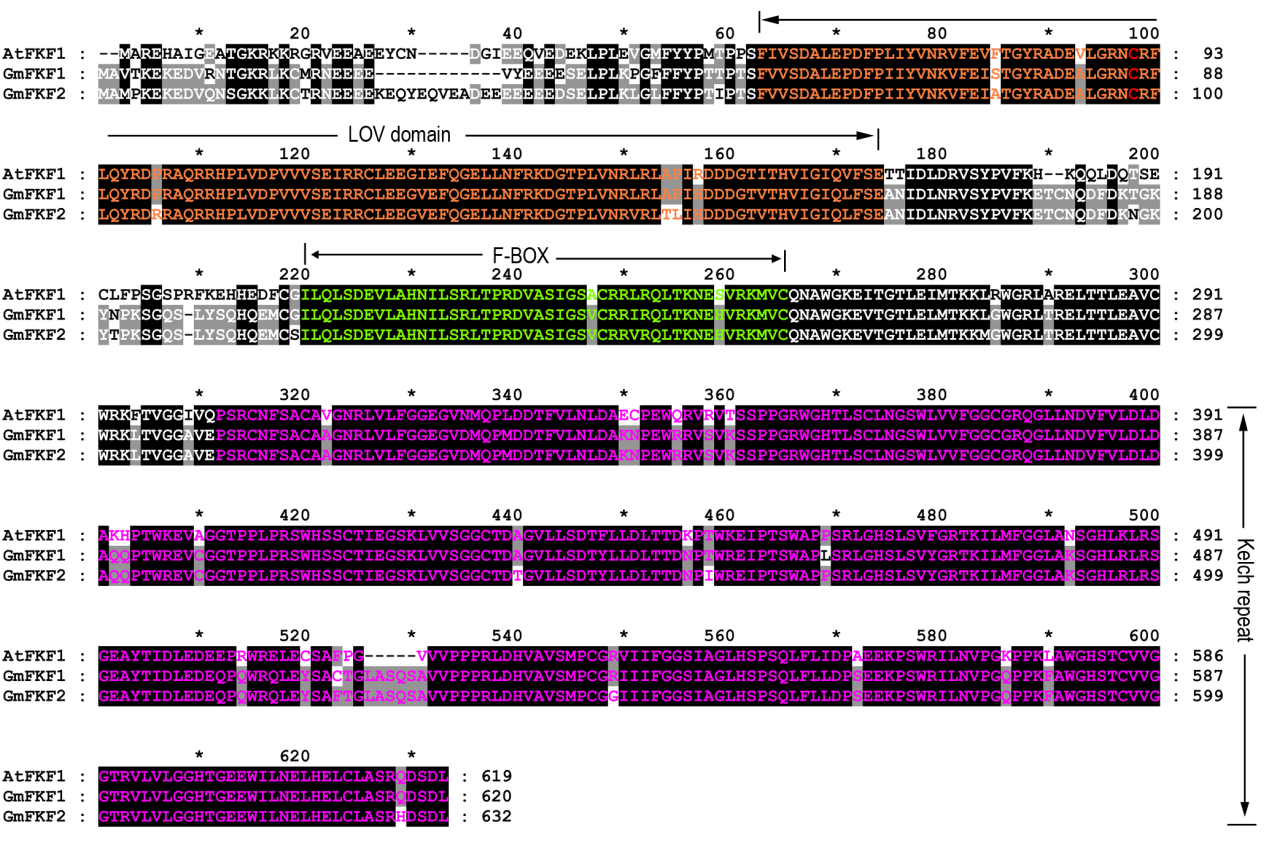
**

**Figure S1 Amino acid sequence alignment of GmFKFs (*Glycine max*) and AtFKF1 (*Arabidopsis thaliana*).** Identical and similar amino acids were indicated in black-shaded and grey-shaded, respectively. The LOV domain, F-box motif, and Kelch repeats were highlighted in yellow fonts, green fonts and pink fonts, respectively.


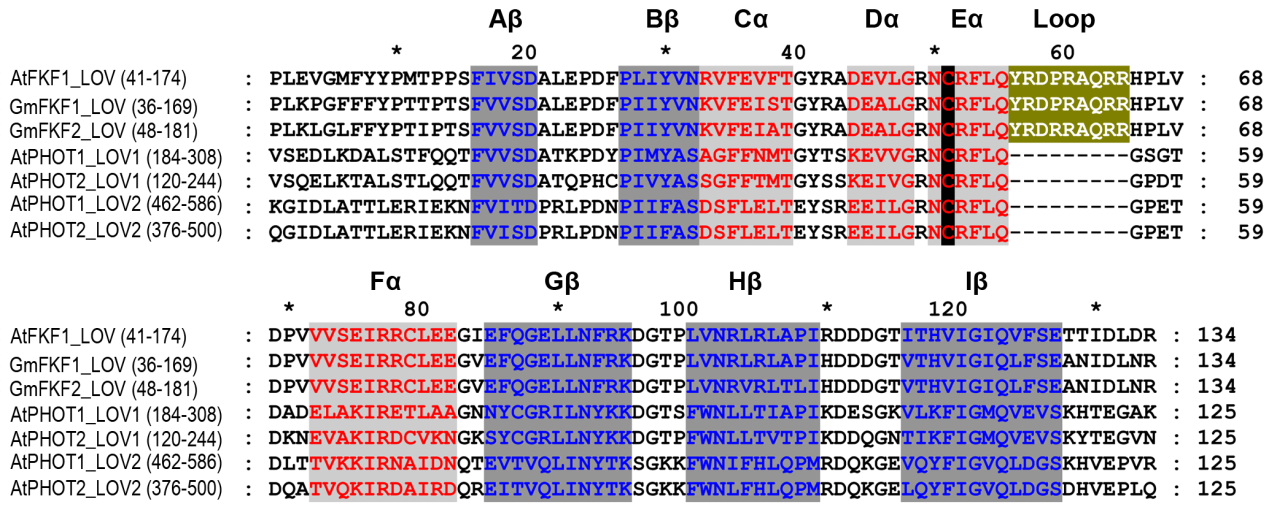


**Figure S2 Comparison of conserved amino acid sites among several LOV domains.** These LOV domains were from *Glycine max* FKF1 and FKF2, *Arabidopsis thaliana* FKF1, PHOT1_LOV1, PHOT1_LOV2, PHOT2_LOV1 and PHOT2_LOV2. The conserved cysteines were highlighted in black background. The loop regions of GmFKF1/GmFKF2/AtFKF1 LOV were in white fonts and green-shaded. The predicted secondary structure elements of α-helices and β-strands were respectively indicated in red fonts and blue fonts. The numbers in the brackets indicated the amino acid positions these LOV domains started and ended.


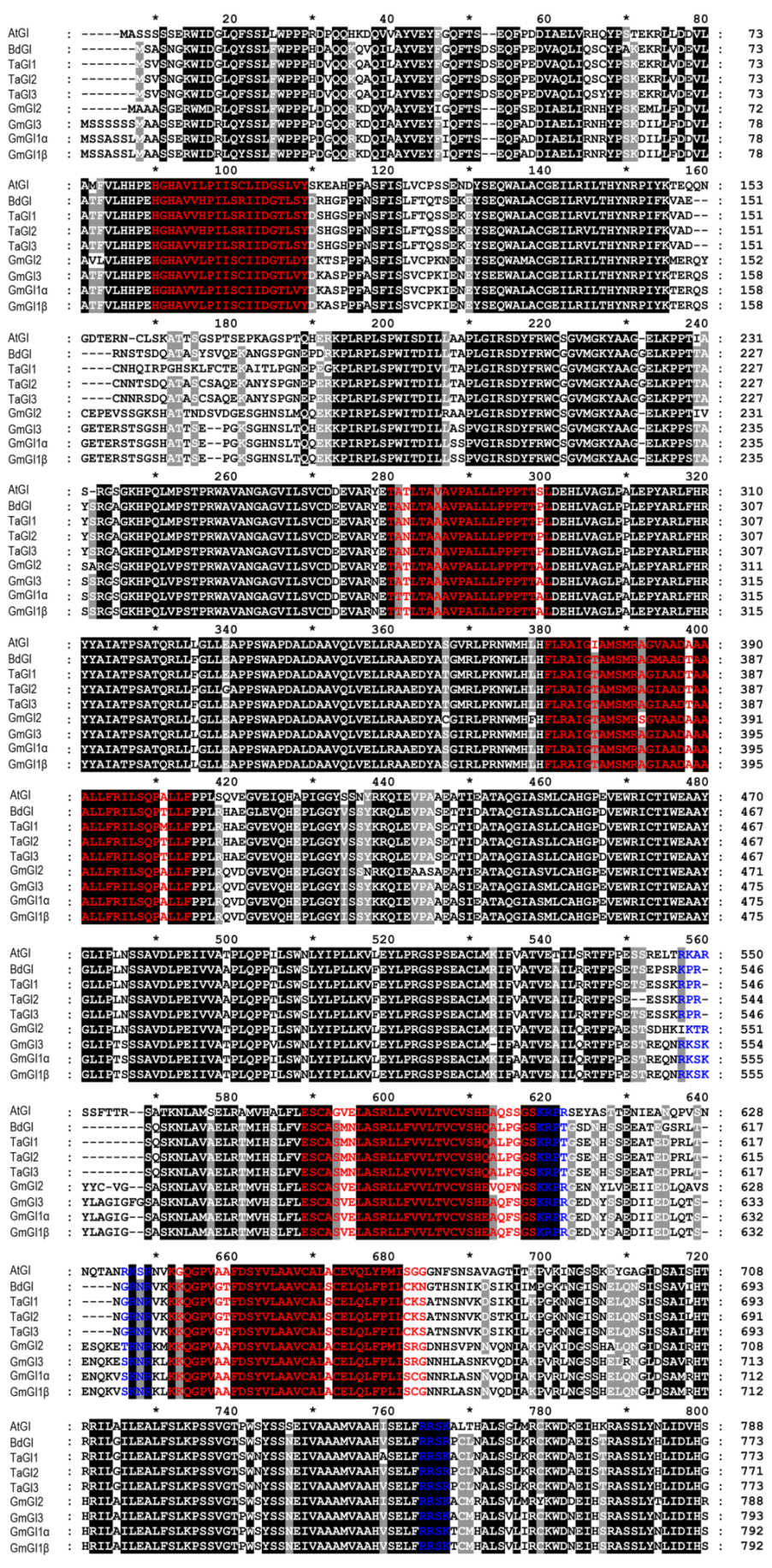


**Figure S3 Alignment of conserved amino acid sites in GI proteins from several species.** These GI proteins included Glycine max GI (GmGIs), Arabidopsis thaliana GI (AtGI), Triticum astivum L. GI (TaGIs) and Brachypodium distachyon GI (BdGI). The black and grey regions represented identical and similar amino acids, respectively. The red region represented transmembrane domains and the blue region represented NLS-like (nuclear localization signals) motifs.


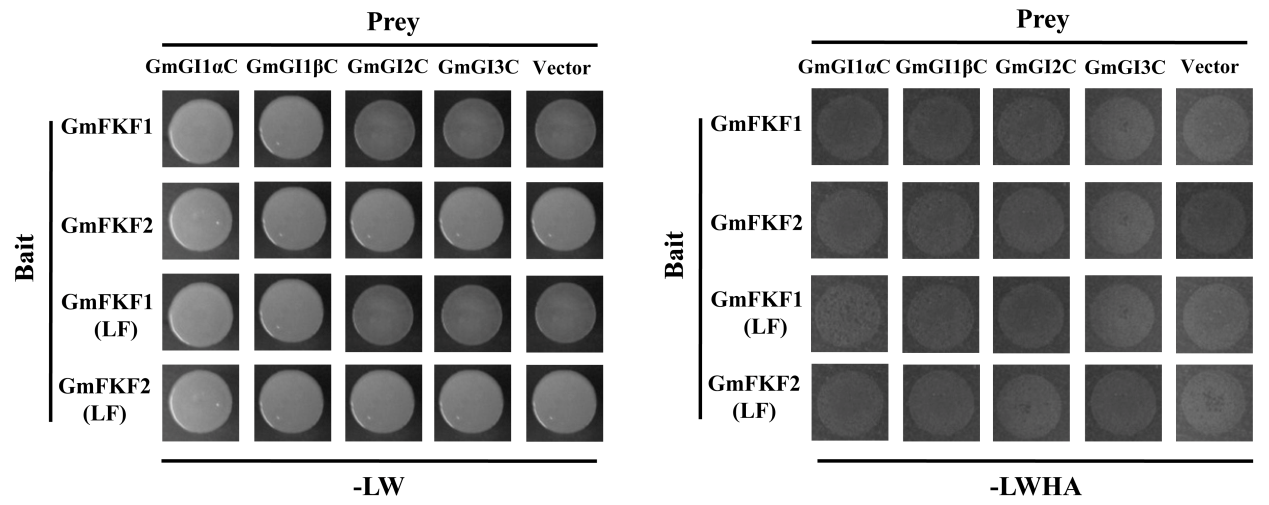


**Figure S4 Interactions between GmGI C-terminal and GmFKFs or GmFKF (LF) truncated proteins in yeast.** GmFKFs (LF) included the LOV domain and F-box motif of GmFKF1 and GmFKF2. GmGI C-terminal were fused to the GAL4 activation domain (Prey). GmFKFs and GmFKFs (LF) were fused to the GAL4 DNA binding domain (Bait). The empty vector only contained the activation domain (AD). -LW, synthetic dropout (SD) yeast growth medium lacking leucine and tryptophan; -LWHA, SD medium lacking leucine, tryptophan, histidine, and adenine.


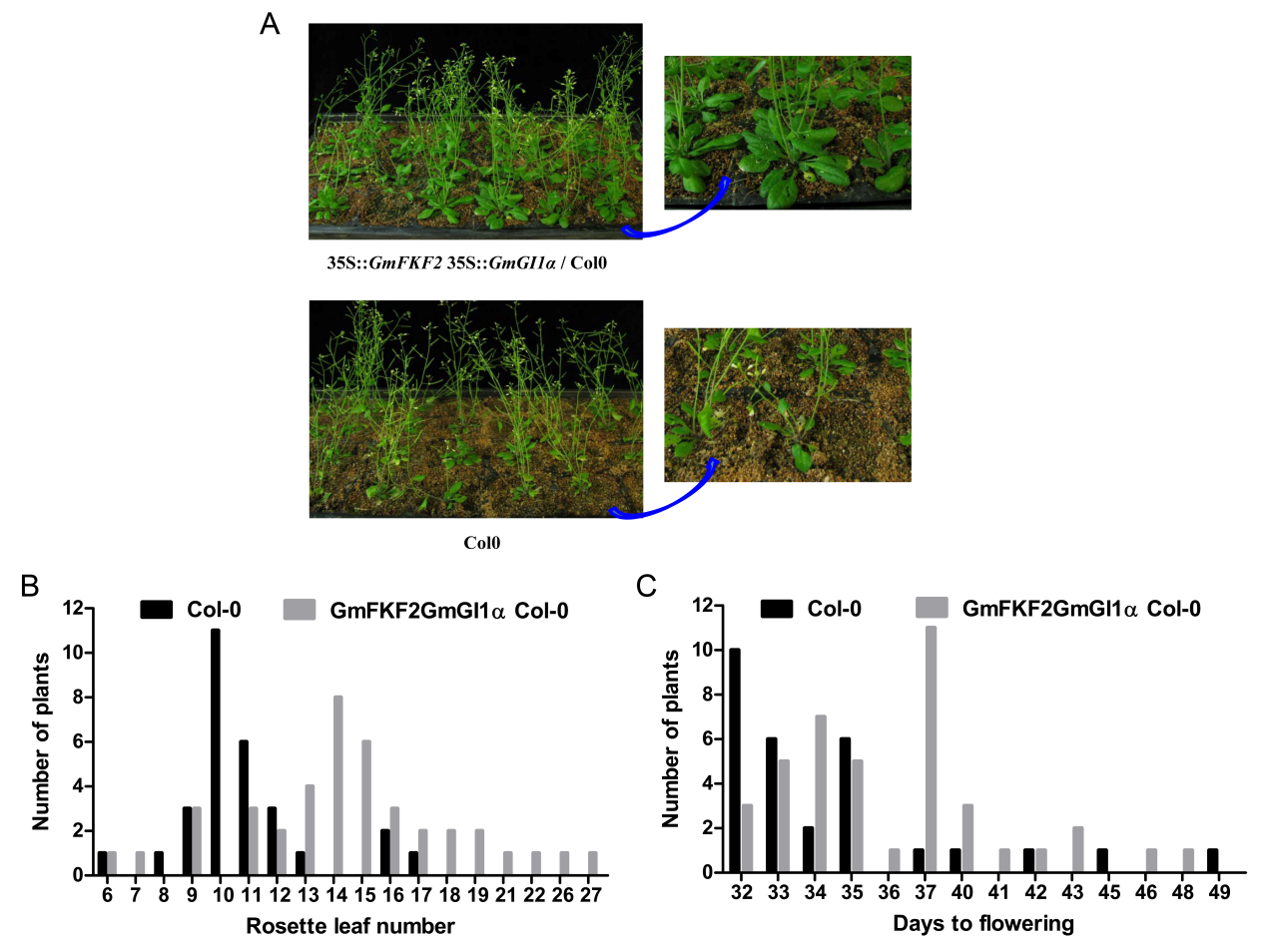


**Figure S5 Phenotypes of *GmGI1α* and *GmFKF2* co*-*overexpressing lines in Arabidopsis Col under LD conditions.** The double transgenic plants exhibited more vigorous growth and senescence retardation and produced more rosette leaves (A). Comparison of the rosette leaf number (B) and the days to flowering (C) between the double transgenic plants and the control plants.
